# The Diagnostic Values of Pretreatment Serum Inflammation Markers and Lipoprotein in Men With Total Prostate-Specific Antigen Between 4 and 10 ng/ml

**DOI:** 10.3389/fmed.2020.576812

**Published:** 2020-11-04

**Authors:** Guangping Li, Zhenhua Shang, Yihao Liu, Hao Yan, Tongwen Ou

**Affiliations:** Department of Urology, Xuanwu Hospital Capital Medical University, Beijing, China

**Keywords:** prostate cancer, inflammation markers, lipoprotein, lipid, prostate biopsy

## Abstract

**Background:** The purpose of this study was to analyze the values of pretreatment serum inflammation markers, lipid, and lipoprotein for predicting the pathological results in men with total prostate-specific antigen between 4 and 10 ng/ml.

**Materials and method:** A total of 611 eligible patients diagnosed with total prostate-specific antigen between 4 and 10 ng/ml and who received a transrectal ultrasound-guided prostate biopsy between January 2014 and December 2019 were included in our study. All the patients were divided into groups according to their pathological results and we collected the data of their pretreatment indicators of the blood routine and biochemistry.

**Results:** The pathological results from prostate biopsies from 160 patients with prostate cancer and 451 patients with benign lesions. Age and total prostate-specific antigen values were significantly higher in patients with prostate cancer than those with benign lesions (*P* < 0.05). Red blood cell, platelet count, prealbumin, and triglyceride were significantly lower in patients with prostate cancer than those with benign lesions. Neutrophil–lymphocyte ratio, platelet–lymphocyte ratio, lymphocyte- monocyte ratio, and apolipoprotein B were lower and apolipoprotein A-I was higher in the prostate cancer group than in the benign lesions group but not significantly (*P* > 0.05). Multivariate logistic regression revealed that age and total prostate-specific antigen could be independent predictors for pathological results (OR, 1.064, 95%CI, 1.031–1.098, *P* < 0.001; OR, 1.232, 95%CI, 1.061–1.429, *P* = 0.006).

**Conclusion:** Higher age and total prostate-specific antigen were closely related to the pathological results. Prospective studies conducted with a large number of patients are needed to evaluate the diagnostic value of non-invasively pretreatment serum inflammation markers and lipoprotein for predicting the pathological results in men with total prostate-specific antigen between 4 and 10 ng/ml.

## Introduction

In 2018, it was estimated that there were 1,276,106 new cases of prostate cancer (PCa), accounting for ~7.1% of all new cases for 36 types of cancer; and 358,989 associated deaths, accounting for ~3.8% of all deaths for 36 types of cancer worldwide. As a result, PCa has become the second most frequent cancer and the fifth leading cause of cancer death in men (1). In recent decades, advanced PCa incidence and disease-specific mortality have been reduced by the diagnosis of asymptomatic prostate cancer by using the test of prostate specific antigen (PSA) (2).

PSA is a glycoprotein secreted by the cells from the prostate gland (3). The PSA test was first available commercially as a screening option in the late 1980's and approved by the FDA in the 1990's (4). By 2001, ~75% of men over 50 years old had undergone PSA testing. According to the National Institute for Health and Care Excellence (NICE) guidelines on prostate cancer 2019, the PSA test remained as the most useful biomarker in the screening for prostate cancer (5). However, when total prostate specific antigen (tPSA) concentrations were between 4 and 10 ng/ml, also called “a gray zone,” only 15.2% of patients had positive prostate biopsies, leading to over-diagnosis and over-treatment (6). Whether prostate biopsies are necessary for patients in gray zone has become a controversial question. Therefore, we need to find some other indicators to provide stronger evidence on whether to perform a prostate biopsy.

Many studies found the potential diagnostic values of neutrophil-lymphocyte ratio (NLR), platelet-lymphocyte ratio (PLR), lymphocyte- monocyte ratio (LMR), and apolipoprotein A-I (Apo A-I) as additional predictable markers for cancers including cervical cancer and bladder cancer (7, 8). For PCa, there was a study showing that the biomarkers of NLR and PLR were significantly different between PCa and benign prostatic hyperplasia (BPH) and a Swedish AMORIS study found that low high-density lipoprotein–cholesterol (HDL), Apo A-I, high triglyceride (TG)/HDL, and low-density lipoprotein–cholesterol (LDL)/HDL were inversely associated with PCa risk (9, 10).

In this study, we examined the association between the levels of pretreatment serum inflammation markers, lipid, lipoprotein, and pathological results to analyze the values of these indicators for predicting the pathological results in men with tPSA between 4 and 10 ng/ml.

## Materials and Methods

### Patients

A total of 611 patients diagnosed with tPSA between 4 and 10 ng/ml who had subsequently received a transrectal ultrasound-guided prostate biopsy at the Department of Urology, Xuanwu Hospital Capital Medical University between January 2014 and December 2019 were included in the study. According to the pathological results, all patients were divided into two groups, 160 patients in the group of prostate cancer and 451 patients in the group of benign lesions.

All pretreatment indicators including age, body mass index (BMI), systolic blood pressure (SBP), diastolic blood pressure (DBP), tPSA, white blood cell (WBC), red blood cell (RBC), hemoglobin (HGB), lymphocyte count (LYMPH), monocyte count (MONO), neutrophil count (NEUT), platelet count (PLT), total protein (TP), albumin (ALB), globulin (GLB), prealbumin (PAB), alkaline phosphatase (ALP), glucose (GLU), TG, total cholesterol (TC), HDL, LDL, Apo A-I, and apolipoprotein B (Apo B) were made available. Patients with any systemic infectious diseases and previous or coexisting malignant tumors were excluded.

NLR and PLR referred to the ratio of NEUT to LYMPH and the ratio of PLT to LYMPH, respectively. LMR referred to the ratio of LYMPH to MONO. A/G was the ratio of ALB to GLB. We also calculated the ratios of LDL/HDL, Apo B/Apo A-I, TG/HDL, and TC/HDL. Clinicopathological characteristics also included histories of hypertension, coronary heart disease, diabetes, and hyperlipidemia. All enrolled patients joined this study voluntarily and signed informed consent for permission to use their data on blood routine tests, biochemical examinations, and pathological results.

### Statistical Analysis

The statistical software of Statistical Product and Service Solutions (SPSS) version 23.0 (Armonk, NY: IBM Corp) and Medcalc Statistical Software version 16.2 (MedCalc Software, Ostend, Belgium) were used to perform the statistical analysis. The distribution of variables was examined by the Shapiro-Wilk test. If the continuous variables fitted normal distribution or fitted log-normal distribution after logarithmic transformation, differences between the two groups were evaluated using *t*-tests. And the differences of abnormal distribution variables were examined using Mann-Whitney *U*-tests. Besides, the Chi-square test was used to analyze categorical variables. Furthermore, the correlation between each variable and pathological result was analyzed by logistical regression analysis and the sensitivity and specificity of each valuable variable were calculated using receiver operating curves (ROC) analyses. In addition, Spearman's rho correlation analysis was used to determine the correlation between the World Health Organization (WHO) groups of PCa following the International Society of Urological Pathology (ISUP) standard and parameters and multivariate analysis between variables and WHO ISUP grades were assessed by using ordinal logistic regression. When the value of *p* < 0.05, the result was considered to be statistically significant.

## Results

All of the 611 patients included in this study received transrectal ultrasound-guided prostate biopsies. According to the pathological results, PCa was found in 160 (26.19%) patients and benign lesions were found in 451 (73.81%) patients. Of these patients with benign lesions, 382 were diagnosed as benign prostatic hyperplasia, and 69 as prostatitis. According to the Gleason scores reported from the pathological results, patients with PCa were classified into five grades following the ISUP standard summarized in the 2019 WHO guidelines. A total of 68 patients were in ISUP grade group 1 (Gleason score 3+3=6). A total of 41 were in ISUP grade group 2 (Gleason score 3+4=7). Twelve were in ISUP grade group 3 (Gleason score 4+3=7). A total of 24 were in ISUP grade group 4 (Gleason score 4+4=8, 3+5=8, 5+3=8) and 15 were in ISUP grade group 5 (Gleason score 5+4=9, 4+5=8, 5+5=10). Considering the number of patients in groups 3–5 were less than group 1 and group 2, we combined the later three groups into one group.

The median ages of the PCa group and benign lesions group was 72.0 (Inter-Quartile Range [IQR], 67.0–78.0) and 67.0 (IQR, 61.0–75.0) years old, respectively (*P* < 0.001) and the clinicopathological characteristics between two groups are shown in Table 1. For our study, compared with a median of 5.51 ng/ml (IQR, 4.64–6.97 ng/ml) for tPSA in the group of benign lesions, the patients with PCa had a significantly higher tPSA (median, 6.13, IQR, 5.02–7.63 ng/ml, *P* = 0.001). The medians of RBC, PLT, PAB, and TG were 4.50 × 109/L (IQR, 4.20–4.82 × 109/L), 197.0 × 109/L (IQR, 160.5–228.0 × 109/L), 227.00 mg/L (IQR, 202.25–265.00 mg/L), and 1.10 mmol/L (IQR, 0.82–1.49 mmol/L), respectively, in patients diagnosed with PCa, significantly lower than those with benign lesions (median, 4.62, IQR, 4.28–4.86 × 109/L *P* = 0.024; median, 199.0, IQR, 169.0, 234.0 × 109/L *P* = 0.041; median, 242.00, IQR, 211.00–272.75 mg/L, *P* = 0.017; median, 1.22, IQR, 0.91–1.62 mmol/L, *P* = 0.034). Besides, 84 (52.5%) patients with PCa and 192 (42.6%) patients with benign lesions had a history of hypertension (*P* = 0.030). A total of 34 (21.3%) patients with PCa and 50 (11.1%) patients with benign lesions had a history of coronary heart disease (*P* = 0.001). The differences were statistically significant. However, there were no significant differences in NLR (*P* = 0.228), PLR (*P* = 0.101), LMR (*P* = 0.748), HDL (*P* = 0.433), LDL (*P* = 0.472), Apo A-I (*P* = 0.173), Apo B (*P* = 0.641), LDL/HDL (*P* = 0.925), Apo B/Apo A-I (*P* = 0.796), TG/HDL (*P* = 0.107), and TC/HDL (*P* = 0.402) between the two groups (Table 1).

**Table 1:** Clinicopathological characteristics between the two groups.

**Characteristic**	**PCa group**	**Benign lesions group**	***P*-value**
	***N* = 160**	***N* = 451**	
Age (IQR), years	72.0 (65.0, 78.0)	67.0 (61.0, 75.0)	<0.001
BMI (Mean ± *SD*), kg/m^2^	24.55 ± 3.53	24.99 ± 3.07	0.162
SBP (IQR), mmHg	127.0 (120.0, 130.0)	120.0 (120.0, 132.0)	0.927
DBP (IQR), mmHg	80.0 (70.0, 80.0)	80.0 (70.0, 80.0)	0.882
tPSA (IQR), ng/ml	6.13 (5.02, 7.63)	5.51 (4.64, 6.97)	0.001
**Blood cell counts, median (IQR)**			
WBC, ×10^9^/L	5.66 (4.78, 6.94)	5.83 (4.89, 6.84)	0.401
RBC, ×10^9^/L	4.50 (4.20, 4.82)	4.62 (4.28, 4.86)	0.024
HGB, g/L	139.0 (130.0, 147.0)	141.0 (133.0, 149.0)	0.095
LYMPH, ×10^9^/L	1.82 (1.42, 2.24)	1.78 (1.45, 2.17)	0.852
MONO, ×10^9^/L	0.41 (0.33, 0.51)	0.40 (0.32, 0.50)	0.322
NEUT, ×10^9^/L	3.31 (2.52, 4.14)	3.30 (2.72, 4.13)	0.183
PLT, ×10^9^/L	197.0 (160.5, 228.0)	199.0 (169.0, 234.0)	0.041
**Systemic inflammatory response parameters, Mean** **±** **SD**			
NLR	1.26 ± 0.16	1.28 ± 0.18	0.228
PLR	3.03 ± 0.16	3.05 ± 0.16	0.101
LMR (IQR)	4.35 (3.58, 5.55)	4.38 (3.50, 5.63)	0.748
**Blood biochemistry, median (IQR)**			
TP, g/L	63.04 (60.06, 66.62)	62.95 (59.94, 66.86)	0.600
ALB, g/L	39.03 (36.99, 41.06)	39.25 (37.32, 41.64)	0.454
GLB, g/L	23.82 (21.70, 26.47)	23.87 (21.58, 26.28)	0.457
A/G	1.65 (1.48, 1.81)	1.66 (1.47, 1.87)	0.357
PAB, mg/L	227.00 (202.25, 265.00)	242.00 (211.00, 272.75)	0.017
ALP, IU/L	60 (50, 69)	60 (52, 72)	0.408
GLU, mmol/L	5.09 (4.65, 5.75)	5.04 (4.66, 5.59)	0.681
TG, mmol/L	1.10 (0.82, 1.49)	1.22 (0.91, 1.62)	0.034
TC, mmol/L	4.04 (3.57, 4.59)	4.07 (3.49, 4.62)	0.658
HDL, mmol/L	1.28 (1.06, 1.50)	1.22 (1.00, 1.44)	0.433
LDL, mmol/L	2.54 (2.04, 3.07)	2.48 (1.95, 2.93)	0.472
Apo A-I, g/L	1.25 (1.09, 1.37)	1.20 (1.04, 1.39)	0.173
Apo B, g/L	0.76 (0.65, 0.91)	0.75 (0.63, 0.88)	0.641
LDL/HDL	1.98 (1.50, 2.66)	2.03 (1.52, 2.60)	0.925
Apo B/Apo A-I	0.62 (0.49, 0.77)	0.63 (0.49, 0.79)	0.796
TG/HDL	0.89 (0.56, 1.24)	0.95 (0.65, 1.51)	0.107
TC/HDL	3.29 (2.61, 3.93)	3.31 (2.70, 4.05)	0.402
Ca, mmol/L	2.19 (2.13, 2.26)	2.19 (2.11, 2.26)	0.342
P, mmol/L	1.02 (0.95, 1.12)	1.01 (0.92, 1.13)	0.424
**Other diseases**, ***n*** **(%)**			
**Hypertension**			
Yes	84/160 (52.5%)	192/451 (42.6%)	0.030
No	76/160 (47.5%)	259/451 (57.4%)	
**Coronary heart disease**			
Yes	34/160 (21.3%)	50/451 (11.1%)	0.001
No	126/160(78.7%)	401/451(88.9%)	
**Diabetes**			
Yes	36/160 (22.5%)	79/451 (17.5%)	0.166
No	124/160 (77.5%)	372/451 (82.5%)	
**Hyperlipidemia**			
Yes	10/160 (6.3%)	19/451 (4.2%)	0.298
No	150/160 (93.7%)	432/451 (95.8%)	

*PCa, prostate cancer; IQR, Inter-Quartile Range; BMI, body mass index; SD, standard deviation; SBP, systolic blood pressure; DBP, diastolic blood pressure; tPSA, total prostate specific antigen; WBC, white blood cell; RBC, red blood cell; HGB, hemoglobin; LYMPH, lymphocyte count; MONO, monocyte count; NEUT, neutrophil count; PLT, platelet count; NLR, neutrophil–lymphocyte ratio; PLR, platelet–lymphocyte ratio; LMR, lymphocyte- monocyte ratio; TP, total protein; ALB, albumin; GLB, globulin; A/G, albumin/globulin ratio; PAB, prealbumin; ALP, alkaline phosphatase; GLU, glucose; TG, triglyceride; TC, total cholesterol; HDL, high-density lipoprotein–cholesterol; LDL, low-density lipoprotein–cholesterol; Apo A-I, apolipoprotein A-I; Apo B, apolipoprotein B; Ca, calcium; P, phosphorus*.

According to the logistic regression, univariate analysis showed that there were positive correlations between age (odds ratio [OR], 1.054, 95% confidence interval [CI] 1.032–1.076, *P* < 0.001), tPSA (OR, 1.207, 95%CI, 1.080–1.349, *P* = 0.001), the history of hypertension (OR, 1.491, 95%CI, 1.038–2.142, *P* = 0.031), coronary heart disease (OR, 2.164, 95%CI, 1.340–3.495, *P* = 0.002), and the pathological result of PCa. RBC (OR, 0.630, 95%CI, 0.430–0.924, *P* = 0.018) and PAB (OR, 0.996, 95%CI, 0.993–1.000, *P* = 0.043) were significantly and negatively correlated with the pathological result of PCa. In the multivariate analysis, only age (OR, 1.064, 95%CI, 1.031–1.098, *P* < 0.001) and tPSA (OR, 1.232, 95%CI, 1.061–1.429, *P* = 0.006) were significantly independent predictors of the pathological results (Table 2).

**Table 2:** Univariate and multivariate logistic regression to identify the predictive factors of the two groups.

**Characteristic**	**Univariate**			**Multivariate**		
	***P*-value**	**OR**	**95%CI**	***P*-value**	**OR**	**95%CI**
Age	<0.001	1.054	1.032–1.076	<0.001	1.064	1.031–1.098
tPSA	0.001	1.207	1.080–1.349	0.006	1.232	1.061–1.429
RBC	0.018	0.630	0.430–0.924	0.875	0.955	0.536–1.702
PLT	0.119	0.997	0.994–1.001	-		
PAB	0.043	0.996	0.993–1.000	0.825	1.001	0.995–1.006
BMI	0.135	0.957	0.904–1.014	-		
HGB	0.201	0.991	0.978–1.005	-		
NEUT	0.257	0.909	0.770–1.072	-		
NLR	0.105	0.834	0.670–1.039	0.109	0.747	0.522–1.067
PLR	0.083	0.996	0.992–1.000	0.394	0.997	0.990–1.004
LMR	0.351	0.949	0.850–1.059	-		
TG	0.202	0.848	0.659–1.092	-		
Apo A-I	0.177	1.892	0.750–4.773	-		
Apo B	0.897	1.070	0.381–3.007	-		
LDL/HDL	0.989	1.002	0.764–1.314	-		
Apo B/Apo A-I	0.554	0.734	0.264–2.043	-		
TG/HDL	0.187	0.841	0.651–1.087	-		
TC/HDL	0.403	0.905	0.717–1.143	-		
Hypertension	0.031	1.491	1.038–2.142	0.162	1.406	0.872–2.265
Coronary heart disease	0.002	2.164	1.340–3.495	0.147	1.587	0.850–2.963
Diabetes	0.167	1.367	0.877–2.130	-		
Hyperlipidemia	0.301	1.516	0.689–3.333	-		

*OR, odds ratio; CI, confidence interval; tPSA, total prostate specific antigen; RBC, red blood cell; PLT, platelet count; PAB, prealbumin; Apo B, apolipoprotein B; Apo A-I, apolipoprotein A-I; BMI, body mass index; HGB, hemoglobin; NEUT, neutrophil count; NLR, neutrophil–lymphocyte ratio; PLR, platelet–lymphocyte ratio; LMR, lymphocyte- monocyte ratio; TG, triglyceride; LDL, low-density lipoprotein–cholesterol; HDL, high-density lipoprotein–cholesterol*.

**Figure 1:**
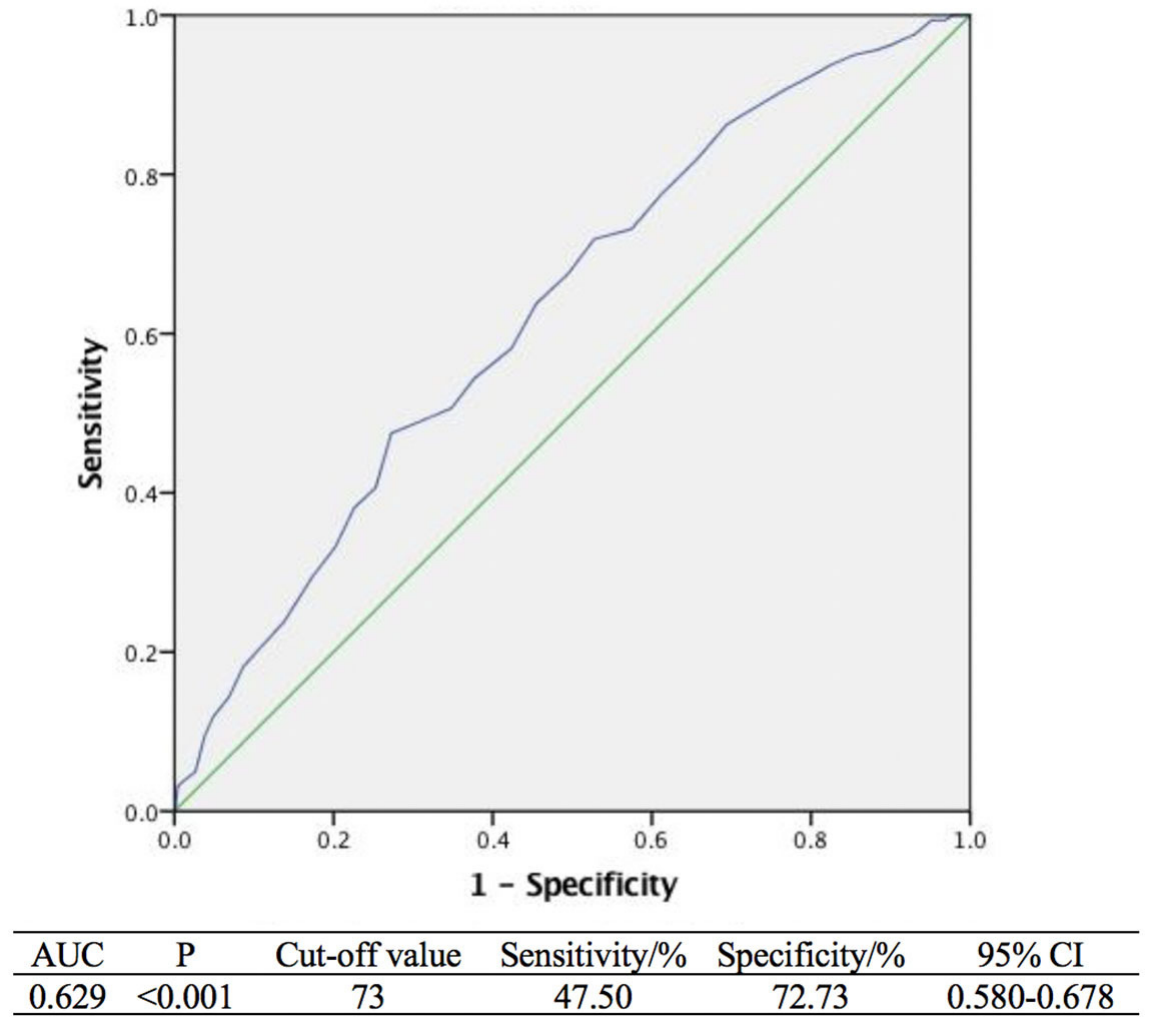
Receiver operating curves analysis of age.

**Figure 2:**
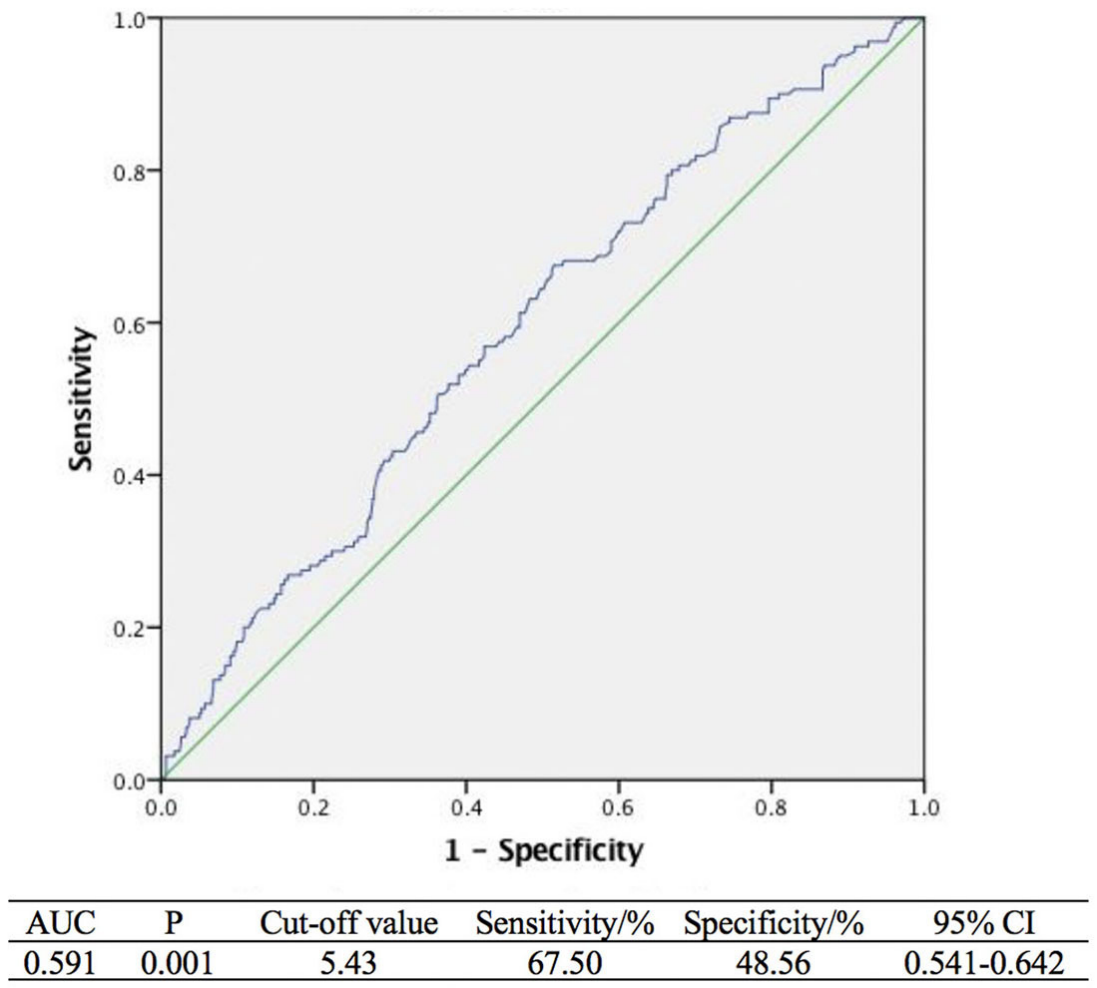
Receiver operating curves analysis of tPSA.

**Figure 3:**
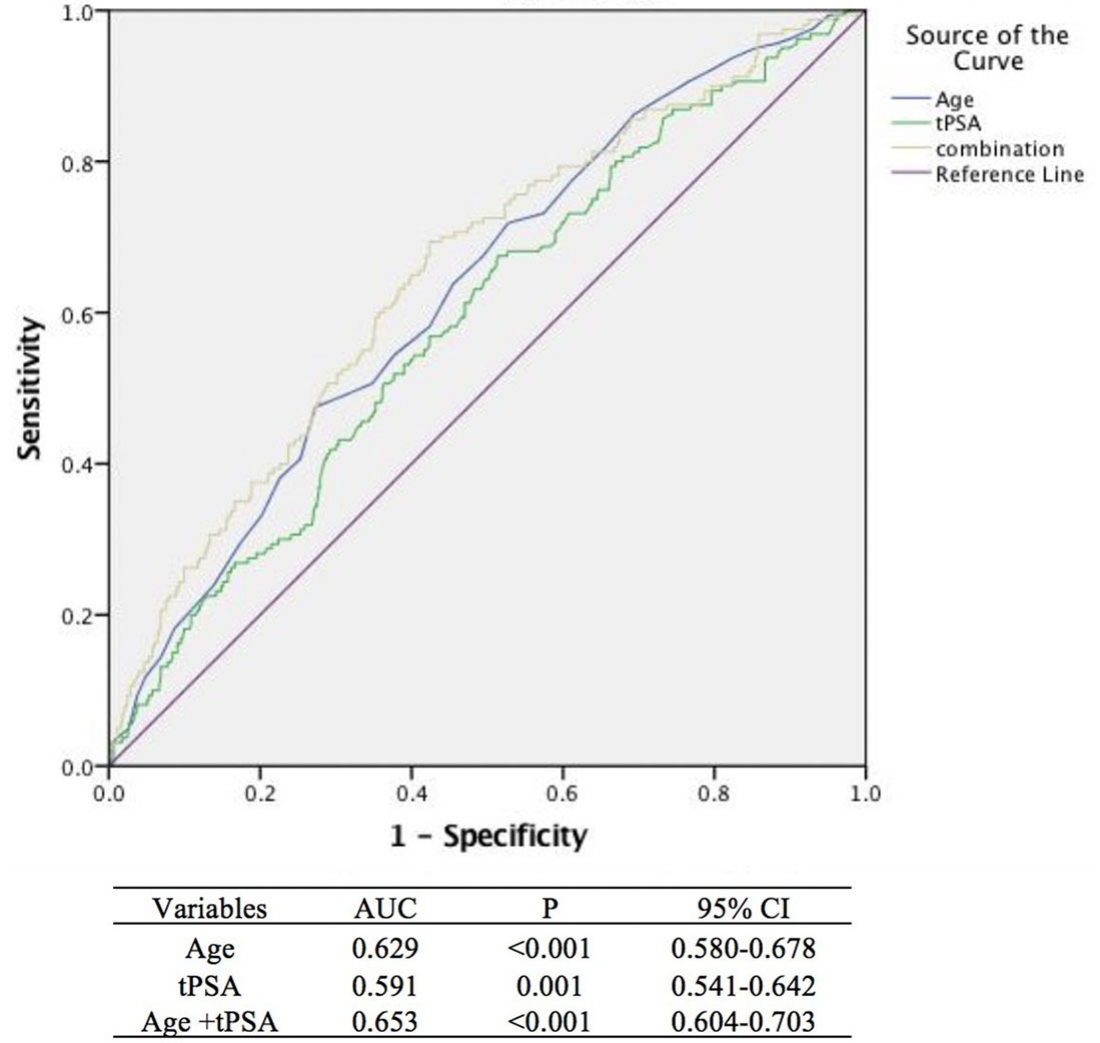
Areas under the curves model analysis of three methods (The blue, green, and yellow lines stand for age, tPSA, and age +tPSA, respectively, with a sensitivity of 69.4% and a specificity of 57.6%).

By analyzing the ROC curve, the sensitivities of age and tPSA were 47.50 and 67.50% and the specificities were 72.73 and 48.56%, respectively. The areas under the curves (AUC) illustrated by the ROC curve were 0.629 (95%CI, 0.580–0.678, *P* < 0.001) and 0.591 (95%CI, 0.541–0.642 *P* = 0.001) with the optimal cut-off values of 73 years old and 5.43 ng/ml. Furthermore, the ROC curve of multivariable analysis revealed that the AUC of the combination of age and tPSA was larger than their simple effects (AUC, 0.653>0.629, 0.591, *P* < 0.001) (Figures 1–3).

Table 3 shows the correlation between the WHO ISUP grades and parameters. Although the p-values of HGB, LMR, and history of hyperlipidemia were <0.05, based on the values of Spearman's rho, there was only a weak correlation between ISUP grades and a history of hyperlipidemia (Spearman's rho = 0.211, *p* = 0.008). Furthermore, the multivariate analyses showed that WHO groups were negatively associated with PLR (OR, −0.046, 95%CI, −0.089 - −0.003, *P* = 0.035) and history of hyperlipidemia (OR, −2.993, 95%CI, −4.982 - −1.004, *P* = 0.003) (Table 4).

**Table 3:** The correlation between the ISUP grades of PCa and parameters.

**Parameters**	**Spearman's rho**	***P*-value**	**Parameters**	**Spearman's rho**	***P*-value**
Age	0.094	0.236	PAB	−0.092	0.245
BMI	0.041	0.612	ALP	−0.069	0.388
SBP	0.136	0.087	GLU	0.070	0.379
DBP	0.077	0.332	TG	−0.003	0.970
tPSA	0.002	0.976	TC	0.072	0.363
WBC	−0.074	0.351	HDL	0.035	0.718
RBC	−0.155	0.050	LDL	−0.007	0.943
HGB	−0.179	0.024	Apo A-I	−0.093	0.338
LYMPH	−0.062	0.437	Apo B	0.030	0.759
MONO	0.074	0.360	LDL/HDL	−0.004	0.964
NEUT	−0.079	0.324	Apo B/Apo A-I	0.098	0.309
PLT	0.008	0.915	TG/HDL	−0.068	0.480
NLR	0.019	0.813	TC/HDL	−0.011	0.909
PLR	0.054	0.497	Ca	−0.032	0.689
LMR	−0.164	0.040	P	0.083	0.296
TP	−0.087	0.275	Hypertension	−0.080	0.322
ALB	−0.118	0.138	Coronary heart disease	0.028	0.728
GLB	−0.009	0.913	Diabetes	−0.099	0.215
A/G	−0.056	0.478	Hyperlipidemia	0.211	0.008

*ISUP, International Society of Urological Pathology; PCa, prostate cancer; BMI, body mass index; SBP, systolic blood pressure; DBP, diastolic blood pressure; tPSA, total prostate specific antigen; WBC, white blood cell; RBC, red blood cell; HGB, hemoglobin; LYMPH, lymphocyte count; MONO, monocyte count; NEUT, neutrophil count; PLT, platelet count; NLR, neutrophil–lymphocyte ratio; PLR, platelet–lymphocyte ratio; LMR, lymphocyte- monocyte ratio; TP, total protein; ALB, albumin; GLB, globulin; A/G, albumin/globulin ratio; PAB, prealbumin; ALP, alkaline phosphatase; GLU, glucose; TG, triglyceride; TC, total cholesterol; HDL, high-density lipoprotein–cholesterol; LDL, low-density lipoprotein–cholesterol; Apo A-I, apolipoprotein A-I; Apo B, apolipoprotein B; Ca, calcium; P, phosphorus*.

**Table 4:** Multivariate analyses between WHO ISUP grades and parameters.

**Parameters**	**Wald**	***P*-value**	**OR**	**95%CI**
Age	2.114	0.146	−0.047	−0.111-0.016
tPSA	2.914	0.088	0.247	−0.037-0.530
RBC	0.178	0.673	−0.329	−1.854-1.197
PLT	3.051	0.081	0.023	−0.003-0.049
PAB	1.206	0.272	0.006	−0.004-0.015
BMI	2.474	0.116	−0.118	−0.264-0.029
HGB	1.180	0.277	−0.032	−0.089-0.026
NEUT	3.510	0.061	−1.358	−2.778-0.063
NLR	2.691	0.101	1.987	−0.387-4.360
PLR	4.468	0.035	−0.046	−0.089 - −0.003
LMR	3.635	0.057	−0.380	−0.771-0.011
TG	0.318	0.573	0.684	−1.693-3.062
Apo A-I	0.008	0.928	−0.231	−5.229-4.767
Apo B	0.062	0.804	−1.242	−11.045-8.561
LDL/HDL	1.124	0.289	−1.423	−4.053-1.207
Apo B/Apo A-I	0.869	0.351	5.953	−6.565-18.472
TG/HDL	1.121	0.290	−1.350	−3.849-1.149
TC/HDL	0.218	0.641	0.607	−1.941-3.154
Hypertension	2.015	0.156	0.649	−0.247-1.545
Coronary heart disease	1.059	0.303	-0.609	−1.768-0.550
Diabetes	0.449	0.503	0.366	−0.704-1.435
Hyperlipidemia	8.702	0.003	-2.993	−4.982 - −1.004

*WHO, World Health Organization; ISUP, International Society of Urological Pathology; OR, odds ratio; CI, confidence interval; tPSA, total prostate specific antigen; RBC, red blood cell; PLT, platelet count; PAB, prealbumin; Apo B, apolipoprotein B; Apo A-I, apolipoprotein A-I; BMI, body mass index; HGB, hemoglobin; NEUT, neutrophil count; NLR, neutrophil–lymphocyte ratio; PLR, platelet–lymphocyte ratio; LMR, lymphocyte- monocyte ratio; TG, triglyceride; LDL, low-density lipoprotein–cholesterol; HDL, high-density lipoprotein–cholesterol*.

## Discussion

Many patients with rising tPSA had the increased likelihood of PCa than those with normal tPSA and diagnostic results had to be confirmed by the pathology. However, for the patients with tPSA in the gray zone, between 4 and 10 ng/ml, only a few patients had positive pathological results, leading to unnecessary biopsies. These unnecessary invasive biopsies not only substantially influenced the patients physically and mentally but also increased the familial and social burden (11). Therefore, it was required that better diagnostic methods be introduced to find additional indicators to provide stronger evidence for performing diagnostic biopsies in patients with tPSA between 4 and 10 ng/ml to avoid over-treatment.

The Perdana et al. study reviewed the risk factors of PCa and found that the risk increased significantly in black men aged over 40 and white men aged over 50 without family history of the cancer (12). The Miotto et al. study found that serum tPSA level could be the basis of selecting transrectal ultrasound-guided prostate biopsy as a treatment (13). Now, tPSA has been widely used clinically in the diagnosis of prostate cancer. From our study, the basic characteristics of age and tPSA were statistically significantly different between PCa and benign lesions. Both of them were independent risk factors and positively correlated to the result of pathology.

The Li et al. study indicated that the variable of PLR was statistically significantly higher in patients with PCa than those with BPH and healthy participants. Besides, PLR, as an independent risk factor, had a close relationship with PCa (14). The Adhyatma et al. study revealed that NLR was significantly elevated in PCa patients and could be used as a biomarker to predict the results of a prostate biopsy. However, in the result, although significant difference was shown in PLR between patients with PCa and those with BPH, PLR was not an independent risk factor from the multivariate analysis (15). There were two related studies, the Kaynar et al. study and the Caglayan et al. study, for evaluating the diagnostic values of the indicators of the blood routine in patients with tPSA between 4 and 10 ng/ml, which mainly analyzed the variables of inflammation markers, including NLR, PLR, and LMR. The first study showed that there was only PSA in no less than the 10 ng/ml groups, the difference of PLR was statistically significant. And there was not any significant difference between the malignant group and the benign group in age, NLR, and mean prostate volume (9). From the results of the other study, similarly, the differences between PCa and benign prostatic hyperplasia (BPH) or prostatitis were not statistically significant in NLR and PLR, but the value of LMR in patients with PCa was higher than those with BPH or prostatitis, and the difference was statistically significant (16).

Consistent with the results from the above two studies, the present study showed there were not statistically significant differences in NLR and PLR, two inflammatory markers, based on the pathological results. However, controversial to the study performed by Caglayan et al., the significant difference was also not observed in LMR between the patients with PCa and those with benign lesions (9, 16). This could be attributed to racial difference, population diversity, and the possible coexistence of PCa and BPH.

Androgens play an important role in prostatic diseases and are generally different between Turkish patients and Chinese patients (17). Besides, the levels of inflammatory markers, the incidence and prognosis of PCa could be influenced by different diets and environments from different countries (18). Based on an epidemiological study in Turkey, the incidence rate of prostate cancer was 35 cases per 100,000, apparently higher than the 16 cases per 100,000 in China in the same period (19, 20).

Moreover, there were not significant differences in NLR, PLR, and LMR between patients with benign lesions and those with PCa. But the multivariate analyses between ISUP grades and parameters in patients with PCa showed that the WHO ISUP grades were negatively associated with PLR (OR, −0.046, 95%CI, −0.089 - −0.003, *P* = 0.035). This result could possibly be due to epidemiology, histopathology, and molecule biology factors. NLR, PLR, and LMR, as inflammatory indicators, referred to the ratios of neutrophil to lymphocyte, platelet to lymphocyte, and lymphocyte to monocyte. Chronic inflammation was common in prostatic diseases, including both benign conditions and PCa. From the baseline data of a REDUCE trial, of all the prostatic biopsies from 8,224 eligible men with BPH, 77.6% had chronic inflammation, of which, 89.0% was mild, indicating a strong association between BPH and chronic inflammation (21). Chronic inflammation also played an influential role in the process of carcinogenesis by synthesizing reactive oxygen and reactive nitrogen compounds, causing the damage of biomacromolecules, including DNA and proteins (22). In 91.6% patients with PCa, at least one biopsy core with inflammation was found in benign areas demonstrating a strong association between PCa and chronic inflammation (23). The coexistence of BPH and PCa should also be taken into account. Approximately 83.3% patients with PCa had concomitant BPH in the prostate (24).

For blood cell counts, in this study, RBC and PLT were significantly lower in patients diagnosed with PCa than those with benign lesions. RBC was inversely related to the pathological results but not the significantly independent factors. The count of RBC in prostate cancer might be influenced by various factors, including nutritional impairment, chronic inflammation, and potential bone marrow infiltration (25). The Sun et al. study showed that the mean values of RBC and PLT were lower in PCa patients than in the controls and that the difference in RBC was statistically significant (26).

Moreover, many studies evaluated the values of the lipid and lipoprotein parameters in diagnosing and prognosing cancer. Apo A-I, as the principal structural protein of HDL, synthesized in the liver and intestine, has been identified as an independent indicator in diagnosis and prognosis of several cancers (8, 27–29). The Li et al. study concluded that the expression of Apo A-I had a significant relationship between bladder cancer and normal controls, so it could be a potential biomarker in diagnosing bladder cancer (27). Likewise, based on the Sirniö et al. study, serum Apo A-I had potential prognostic value in patients with colorectal cancer (29). Besides, the Van et al. study analyzed the relationship between lipid parameters and the pathological results of PCa and found that low Apo A-I and HDL were statistically significantly associated with PCa (10). The Asare et al. study showed that LDL was significantly higher in a PCa group than a BPH group and had a strong positive association with the pathological results (30).

The present study first evaluated the lipid and lipoprotein parameters, including TP, ALB, GLB, A/G, PAB, TG, TC, HDL, HDL, Apo A-I, Apo B, LDL/HDL, Apo B/Apo A-I, TG/HDL, and TC/HDL in patients with tPSA between 4 and 10 ng/ml for predicting the pathological results. It showed that PAB and TG in patients with PCa were significantly lower than those with benign lesions. The logistic regression found that there was a significantly negative correlation between PAB and the pathologic result of PCa, but PAB was not a significantly independent predictor of PCa. PAB, as a sensitive indicator of nutritional status and metabolic state in clinical practice, is a protein synthesized mainly in the liver (31). In patients with cancer, nutrition disorders, especially malnutrition, accounting for about 79%, was frequently found. The possible cause was that nutrition played an important role in sustaining the normal function of the immune system (32, 33). The Rostenberg et al. study showed the same result that the level of serum PAB decreased significantly in patients of the cancer group than those of the benign diseases groups (34).

Furthermore, this study analyzed the relationship of some disease histories and the risk of PCa. The Navin et al. study evaluated the incidence of hypertension in 3,200 PCa patients and showed that 72% of white patients and 73% of African American patients also suffered from hypertension, indicating that hypertension might be a risk factor in the causation of PCa (35). The Stamatiou et al. study indicated there was a statistically significant relation between the presence of latent prostate cancer and the severity of coronary heart disease (36). In our study, for patients with tPSA between 4 and 10 ng/ml, the incidence of hypertension and CHD were significantly higher in the PCa group than in the benign lesions group, and multivariate analyses between the ISUP grades and parameters in patients with PCa showed that the WHO ISUP grades were negatively associated with a history of hyperlipidemia. These results seemed to correspond with an increase of androgens. Androgens could affect the reabsorption of sodium by activating the renin-angiotensin system (RAS) inappropriately. The main effector of RAS, angiotensin II (Ang II), increased greatly in patients with prostate cancer, leading to hypertension and cardiovascular morbidity (37).

In addition, we first conducted a subgroup analysis on the correlation between the malignant degree of PCa and parameters. However, the result showed that the *p*-values of HGB, LMR, and history of hyperlipidemia were <0.05, however, according to the values of Spearman's rho, there was only a weak correlation between the degree of PCa and history of hyperlipidemia (Spearman's rho = 0.211, *p* = 0.008). This might correspond to the existence of chronic inflammation in the process of carcinogenesis by synthesizing reactive oxygen and reactive nitrogen compounds, causing the damage of biomacromolecules, including DNA and proteins (22, 23).

As a single-center retrospective study, there were some limitations. First, when the patients with tPSA between 4 to 10 ng/ml who met the included criteria were chosen and analyzed in this study, to some extent, there was the possibility of inherent selection bias and analysis bias, affecting the results. Second, although we analyzed age, BMI, tPSA, the history of other diseases, inflammation markers, lipid, and lipoprotein markers, there were also other factors such as smoking history and very low-density lipoprotein (VLDL) that could be closely related to the pathological results. In addition, the sample sizes was still small, which might be one of the reasons causing the difference in LMR between this study and the Caglayan et al. study (16). Thus, a prospective multi-center randomized control trial is needed to validate our results. Finally, considering that many indicators of inflammation, lipid, and lipoprotein markers have shown diagnostic and prognostic values between prostate cancer and benign lesions, further basic laboratory research is needed to figure out their mechanism.

## Conclusions

Higher age and tPSA, as independent factors, were closely related to the pathological results for patients in the gray zone. Prospective studies conducted with large patients are needed to evaluate the diagnostic value of non-invasive pretreatment serum inflammation markers and lipoprotein for predicting the pathological results in men with tPSA between 4 and 10 ng/ml. Moreover, further basic research to clarify the interaction of these parameters and prostate cancer is needed.

## Data Availability Statement

All datasets presented in this study are included in the article/supplementary material.

## Ethics Statement

Written informed consent was obtained from the individual(s) for the publication of any potentially identifiable images or data included in this article.

## Author Contributions

GL: project development, data collection, analysis and interpretation of data, and manuscript writing. ZS, YL, and HY: data collection and analysis and interpretation of data. TO: project development, supervision, and manuscript writing. All authors contributed to the article and approved the submitted version.

## Conflict of Interest

The authors declare that the research was conducted in the absence of any commercial or financial relationships that could be construed as a potential conflict of interest.
